# AcfA Regulates the Virulence and Cell Envelope Stress Response of *Vibrio parahaemolyticus*

**DOI:** 10.3390/microorganisms13010007

**Published:** 2024-12-24

**Authors:** Huan Liu, Huayu Lei, Juanjuan Cao, Zhaobang Xie, Yile Shi, Yanni Zhao

**Affiliations:** 1 School of Food Science and Engineering, Shaanxi University of Science & Technology, No. 6 Xuefu Road, Xi’an 710021, China; 2 Shaanxi Research Institute of Agriculture Products Processing Technology, No. 6 Xuefu Road, Xi’an 710021, China

**Keywords:** *Vibrio parahaemolyticus*, AcfA, virulence, cell envelope stress, biofilm

## Abstract

*Vibrio parahaemolyticus* is a ubiquitous inhabitant of estuarine and marine environments that causes vibriosis in aquatic animals and food poisoning in humans. Accessory colonizing factor (ACF) is employed by *Vibrio* to assist in the colonization and invasion of host cells leading to subsequent illnesses. In this work, Δ*acfA*, an in-frame deletion mutant strain lacking the 4th to the 645th nucleotides of the open reading frame (ORF) of the *acfA* gene, and the complementary strain *acfA*^+^ were constructed to decipher the function of AcfA in *V. parahaemolyticus*. The deletion of *acfA* had no effect on bacterial growth but resulted in a significant reduction in biofilm formation, hemolytic activity, mucus adhesion, and the accumulated mortality of zebrafish, compared to the wild-type strain and the complementary strain *acfA*^+^. Additionally, AcfA was involved in adapting to stressors, such as H_2_O_2_, EDTA, and acid, in *V. parahaemolyticus*. Furthermore, RNA-Seq transcriptome analysis was conducted to identify global gene transcription alterations resulting from deletion of the *acfA* gene. A total of 416 differentially expressed genes were identified in the Δ*acfA* vs. wild-type comparison, with 238 up-regulated genes and 178 down-regulated genes. The expression of genes associated with the type III secretion system, type VI secretion system, and oligopeptide permeases system were significantly reduced, and yet the expression of genes associated with cell envelope biosynthesis and response regulation system were enhanced dramatically in the absence of the *acfA* gene compared to the wild-type strain. These findings suggest that AcfA may play a role in the overall success of pathogenesis and the cell envelope stress response of *V. parahaemolyticus*.

## 1. Introduction

*Vibrio parahaemolyticus* is a halophilic bacterium naturally found in marine and estuarine environments. It has gained significant attention due to its increasing prevalence as a foodborne pathogen [1]. The global distribution of *V. parahaemolyticus* infection varies, with higher prevalence reported in regions with warmer climates and coastal areas. Countries such as Japan, China, the United States, and several European countries have reported outbreaks and cases associated with the consumption of contaminated seafood. *V. parahaemolyticus* causes approximately 45,000 illnesses and 100 deaths in the United States each year, and the number of cases has been increasing in recent years [2]. *V. parahaemolyticus* infections primarily result in gastroenteritis, characterized by symptoms such as watery diarrhea, abdominal cramps, nausea, and vomiting. However, in severe cases, it may lead to septicemia and even death [3]. This bacterium possesses various virulence factors that contribute to its ability to colonize and cause disease in the host, such as extracellular toxins, outer membrane proteins, adhesion systems, secretion systems, the capacity for biofilm formation, and so on [4].

Most pathogens need to be able to thrive both inside and outside of the host. The capacity of different microbes to attach to and colonize abiotic or biotic surfaces is a typical process by which they improve their ability to live in a variety of situations. Accessory colonizing factor (ACF) is employed by *Vibrio* to assist in its colonization and invasion of host cells, leading to subsequent illnesses. ACF was first identified in *V. cholerae* and is encoded by a gene cluster composed of four genes: *acfA*, *acfB*, *acfC*, and *acfD* [5]. Disruption of any of these four acf genes reduces the ability of *V. cholerae* to colonize the intestines of suckling mice. One of the crucial *Vibrio* virulence genes is the *acfA* gene [6,7,8]. In *V. alginolyticus*, the deletion of *acfA* caused a remarkable decrease in motility, extracellular protease activity, expression of virulence factors such as Clp, Fla, and OmpA, and ability to colonize the intestine of pearl gentian grouper, but enhanced biofilm formation. Of note, the LD_50_ value of the Δ*acfA* mutant strain (3.17 × 10^8^ CFU/fish) was higher than that of the wild-type strain and the complementary strain (2.42 × 10^6^ and 3.01 × 10^6^ CFU/fish, respectively) [8]. Furthermore, the Δ*acfA* mutant strain induced a high antibody titer and displayed an excellent immunoprotective effect with a relative percent survival (RPS) of 81.1% in pearl gentian grouper against *V. alginolyticus* infection [9]. Moreover, the recombinant AcfA protein induced an obviously higher antibody titer and showed a high level of protection in *Lutjanus erythropterus* Bloch against infection with six virulent *V. alginolyticus* strains, with RPS values of 95.24%, 85.37%, 83.33%, 90.48%, 90.48%, and 92.68%, respectively [10]. To our knowledge, the function of AcfA in *V. parahaemolyticus* has not yet been explored.

In the present investigation, we focused on the pleiotropic roles of AcfA in the different physiological functions and virulence of *V. parahaemolyticus* RIMD2210633.

## 2. Materials and Methods

### 2.1. Bacterial Strains, Plasmids, and Culture Conditions

The bacterial strains and plasmids used in this study are listed in Table 1. *V. parahaemolyticus* RIMD2210633 was stored at −80 °C in LB broth containing 20% (*v*/*v*) glycerol. The stock culture was propagated in LB broth containing 100 μg/mL ampicillin (*w*/*v*) and then cultured at 37 °C with agitation (200 rpm) for 12 h for further assays. All strains were grown in LB by shaking at 200 rpm for liquid cultivation or on LB plates with 1.5% (*w*/*v*) agar for solid cultivation. *Escherichia coli* growth medium was supplemented with antibiotics according to plasmid characteristics.

### 2.2. Construction of the Mutant ΔacfA Strain and the Complemented Strain acfA^+^

The Δ*acfA* fragment with deletion of the region from the 4th to 645th bases was generated by overlap PCR using the genomic DNA of *V. parahaemolyticus* RIMD2210633 as the template. The Δ*acfA* fragment was then cloned into the suicide plasmid pDM4 and sequentially transformed into *Escherichia coli* DH5α λ pir and SM10 λ pir. Bacterial conjugation was carried out between the wild-type strain of *V. parahaemolyticus* and the recombinant *E. coli* SM10 λ pir strain containing pDM4-Δ*acfA*. The mutant strain, Δ*acfA*, was obtained through two rounds of homologous recombination, using positive and negative selection markers, respectively. For complementation strain construction, the recombinant plasmid, pMMB207-*acfA*, was conjugated into the mutant strain, Δ*acfA*. Isolated colonies were screened using PCR to identify cells in which gene deletion or complementation was successfully achieved. All the mutant and complemented strains were verified by sequencing. The primers used are listed in Table 2.

### 2.3. Measurement of Bacterial Growth

Overnight bacterial cultures, diluted to OD_600_ = 1.0, were inoculated into the LB broth with a 1% (*v*/*v*) inoculation amount, and incubated for 14 h at 37 °C. The optical density was measured at a wavelength of 600 nm every hour.

### 2.4. RNA-Seq Analysis

After being cultivated for an overnight period, the wild-type and Δ*acfA* strains were diluted 1:100 into new LB medium. After 8 h of cultivation, total RNA was extracted using Trizol reagent (Invitrogen, Carlsbad, CA, USA) according to the manufacturer’s instructions. The RNA samples were cleaned of any contaminating genomic DNA (gDNA) using DNase I (Takara, Takyo, Japan) and then sequenced using an Illumina NovaSeq6000 (Illumina Inc., San Diego, CA, USA). Transcripts per million mapped reads (TPM) was utilized to calculate expression levels by RSEM (http://deweylab.github.io/RSEM/) (accessed on 30 March 2024). DESeq2 Bioconductor software Version 1.42.0 was used to characterize the differentially expressed genes with *p*-adjust < 0.05 and an absolute fold change (FC) ≥ 1.5. To identify the functional categories of the DEGs, Gene Ontology (GO) terms and Kyoto Encyclopedia of Genes and Genomes (KEGG) pathway enrichment analyses were conducted. The COG (Cluster of Orthologous Groups of Proteins) protein database was used to determine protein function. All of the data analyses were performed using the online platform of Majorbio Cloud Platform (www.majorbio.com) (accessed on 30 March 2024) from Shanghai Majorbio Bio-Pharm Technology Co., Ltd. (Shanghai, China).

### 2.5. Quantitative Realtime PCR (qRT-PCR)

Total RNA was extracted using Trizol. For reverse transcription polymerase chain reaction (RT-PCR), 1 μg of pure RNA from cultures was added to 5 × All-in-One qRT SuperMix (Vazyme, Xi’an, China) to obtain cDNA. SuperReal Color Premix (SYBR Green) (Vazyme, Xi’an, China) was used for the qRT-PCR assay, and the primers are shown in Table S1. qRT-PCR was carried out on a Bio-Rad CFX Opus Real-time PCR system with at least three independent tests. The genes’ transcriptional levels were standardized to the 16S rRNA level according to the 2^−ΔΔCt^ method [13].

### 2.6. Biofilm Assay

The formation of biofilms was determined by crystal violet (CV) staining. Overnight-cultured cell suspensions were diluted to an OD_600_ of 1.0 and then subcultured into fresh LB broth qt an inoculation volume of 1%. The cultures were incubated at 37 °C with agitation at 120 rpm for 12 h, then stained with 2% (*w*/*v*) crystal violet solution for 5 min and cleaned three times with distilled water. After the tubes were dried, 1 mL of 33% (*v*/*v*) acetic acid was added to dissolve the CV stain. The absorbance of the resultant solution in each tube was determined at 570 nm.

### 2.7. Kanagawa Phenomenon (KP) Test

In total, 2.5 μL of the overnight cultured cell suspensions, diluted to an OD_600_ of 1.0 with LB broth, was spotted onto Wagatsuma agar plates containing 5% rabbit red blood cells (Hopebio, Qingdao, China). After static incubation at 37 °C for 12 h, the radius from the spotting site to the margin of the β-hemolysin zone was measured, and a photograph was taken.

### 2.8. In Vitro Mucus Adhesion Assay

In accordance with previous work by Huang et al. [14], healthy groupers obtained from a local seafood market in the city of Xi’an, Shaanxi province of China, were utilized for mucus preparation. The skin mucus was gathered after washing with sterile PBS (0.01 mol/L, pH 7.2) by scraping the skin’s surface with a plastic spatula to remove the mucus gel layer. This layer was then homogenized in PBS. To eliminate particle elements, the mucus preparations were centrifuged twice (20,000× *g*, 4 °C, 30 min) and then filtered through 0.45 μM and 0.22 μM filters. Using the Bradford method, the mucus samples were adjusted to 1 mg protein/mL PBS.

A total of 100 μL of mucus was equally dispersed over a 20 mm × 20 mm glass slide and dried at room temperature. The mucus-coated glass slides were then loaded with 300 μL of bacterial suspension (OD_600_ = 1.0), cultured for 2 h at 37 °C, and then rinsed five times with PBS. Finally, the bacteria were fixed with 4% (*v*/*v*) methanol for 30 min, stained for 3 min in crystal violet, and counted under a microscope (×1000), with 20 different views chosen.

### 2.9. Scanning Electron Microscopy (SEM) Observation

SEM analysis was carried out in accordance with a method described previously [15]. In brief, *V. parahaemolyticus* cells were collected following centrifugation at 10,000× *g* for 10 min at 4 °C. Prior to SEM examination, the cell pellet was preserved in 2.5% glutaraldehyde at 4 °C overnight. The fixed cells were dehydrated in ethanol (30%, 50%, 70%, and 90%) (*v*/*v*) for 10 min, soaked in 100% (*v*/*v*) isoamyl acetate for 30 min, and dried at 80 °C for 1 h. Microscopy and imaging were performed on a FESEM Zeiss Sigma 300 (Zeiss, Oberkochen, Germany).

### 2.10. Stress Response Assay

Strains were inoculated at a 1:100 ratio from an overnight pre-culture grown in LB and shaken till mid-log phase. They were inoculated into LB broth supplemented with different levels of SDS (0.5%, 0.7% (*w*/*v*)), EDTA (0.04%, 0.06% (*w*/*v*)), or H_2_O_2_ (5 mmol/L, 7 mmol/L, 9 mmol/L). The optical density of the cultures at 600 nm was determined every 0.5 h. Bacterial growth in LB broth served as a control. For the acid sensitivity assay, bacteria were cultured to the same OD_600_ value (0.5), and the cells were collected and subcultured in LB medium at pH = 7 or pH = 5.0 for 1 h. Then, bacteria were subjected to acid stress in pH = 3.5 medium, and an aliquot was taken from each culture at 0 h, 0.5 h, and 1 h. The cell cultures were subjected to five-fold serial dilution (from 5^0^ to 5^−5^), and 2.5 μL of each culture was dripped onto LB plates [16]. The plates were then all incubated at 37 °C. All of the aforementioned experiments were performed at least three times, and one representative experiment’s findings are presented.

### 2.11. Fish Infection Assay

Overnight bacterial cultures were adjusted to the same OD_600_ value (1.0) and then serially diluted in PBS for infection tests in zebrafish. By intramuscular (i.m.) injection, the strains were administered to zebrafish weighing about 1 g at cell densities ranging from 10^4^ to 10^7^ colony-forming units (CFU)/fish, as previously described. Twenty fish were infected with each dosage, and three parallel tests were performed. Over the course of seven days, the number of dead fish was counted, and the LD_50_ value was determined.

### 2.12. Statistical Analyses

Every experiment was carried out in triplicate. Statistical analyses were carried out using GraphPad Prism 7.00 (San Diego, CA, USA), and the results are reported as the mean ± SD (*n* = 3). Unless otherwise specified, differences in means were examined using the Student’s t test. Significant differences were considered as *p* ≤ 0.05.

## 3. Results

### 3.1. Phenotypic Alteration Caused by Deletion of acfA in Vibrio parahaemolyticus

In this investigation, label-free gene deletion based on two rounds of homologous recombination with the suicide plasmid pDM4 was performed to obtain the Δ*acfA* mutant strain. Successfully, an in-frame deletion mutant strain lacking nucleotides 4 to 645 of the ORF of the *acfA* gene was identified and verified by PCR and DNA sequencing. Meanwhile, the complementary strain *acfA*^+^ was also acquired by introducing the recombinant plasmid pMMB207-*acfA* into Δ*acfA* (Figure S1).

**Figure 1:**
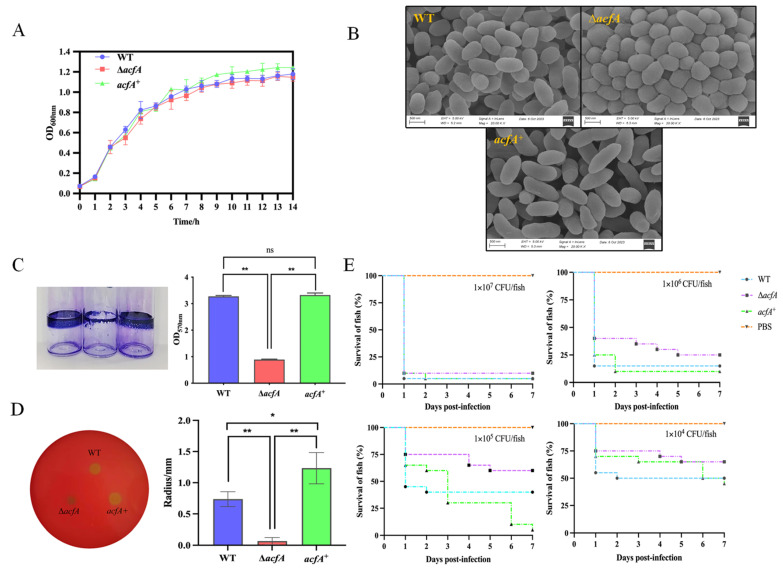
The roles of AcfA in the growth, cell morphology, and virulence of *V. parahaemolyticus*. (**A**) Growth of different *V. parahaemolyticus* strains in LB broth. (**B**) Cell morphology observation of the wild-type (WT), Δ*acfA*, and *acfA*^+^ strains by SEM (the magnification is 20,000×). (**C**) Effect of AcfA on the biofilm formation of *V. parahaemolyticus*. (**D**) Effect of AcfA on the hemolytic activities of different strains, as assessed by the Kanagawa Phenomenon (KP) test. (**E**) Survival of zebrafish infected with different strains at different doses (1 × 10^7^ CFU/fish, 1 × 10^6^ CFU/fish, 1 × 10^5^ CFU/fish, 1 × 10^4^ CFU/fish), with PBS as the control. (* *p* ≤ 0.05, ** *p* ≤ 0.01, ns means not significant).

The growth rates of the wild-type (WT), Δ*acfA*, and *acfA*^+^ strains were assessed in order to understand the function of the *acfA* gene in bacterial reproduction. The findings revealed no discernible difference in the growth of the three strains in LB broth during the testing period of 14 h (Figure 1A), demonstrating that the *acfA* gene has no impact on bacterial growth in rich medium (LB). From SEM observation, the findings revealed that the WT and *acfA*^+^ strains exhibit a short rod shape, while the Δ*acfA* strain rods were widened, and there were signs of rounding (Figure 1B).

As an accessory colonizing factor, AcfA has been proven to be involved in *Vibrio* adhesion. Herein, an in vitro mucus adhesion assay was carried out. The findings demonstrated that *V. parahaemolyticus* can attach to grouper mucus efficiently. Deletion of *acfA* significantly inhibited the bacterial adhesion capacity to grouper mucus, while the *acfA*^+^ strain showed recovered adhesion ability (Table 3). This research revealed that the *acfA* gene plays a pivotal role in adhesion in *V. parahaemolyticus*. Adhesion to a biotic or abiotic surface is the initial step in bacterial biofilm formation. Therefore, we hypothesized that AcfA is essential for biofilm formation in *V. parahaemolyticus*. Crystal violet staining was employed to quantify biofilm formation in the wild-type, Δ*acfA*, and *acfA*^+^ strains. The results indicated that the wild-type strain was capable of forming a massive and dense biofilm on a glass surface, and deletion of the *acfA* gene resulted in a remarkable reduction in biofilm formation. When the mutant strain was complemented with an intact *acfA* gene, the capability of biofilm formation was restored to the level seen in the wild-type strain (Figure 1C). Moreover, the Δ*acfA* strain displayed a dramatic drop in hemolytic activity compared to the wild-type strain. Yet, the *acfA*^+^ strain could form an obvious β-hemolysin zone on Wagatsuma agar plates (Figure 1D). It follows that AcfA plays prominent roles in hemolysin production in *V. parahaemolyticus*. Furthermore, zebrafish were used as an infection model to evaluate and compare the virulence of the three strains to determine the involvement of the *acfA* gene in the pathogenicity regulation of *V. parahaemolyticus*. According to our findings, zebrafish infected with the Δ*acfA* strain exhibited the highest survival rate at each infection dose. For instance, at a dose of 1 × 10^5^ CFU/fish, zebrafish infected with the Δ*acfA* strain showed a higher survival rate (60%) than zebrafish infected with the wild-type strain (40%) or the *acfA*^+^ strain (10%). Additionally, the zebrafish in the group treated with phosphate-buffered saline (PBS) exhibited a 100% survival rate (Figure 1E). The results showed that AcfA positively regulated the pathogenicity of *V. parahaemolyticus* in a zebrafish model.

### 3.2. AcfA Is Essential for Environmental Stress Adaptation in V. parahaemolyticus

**Figure 2:**
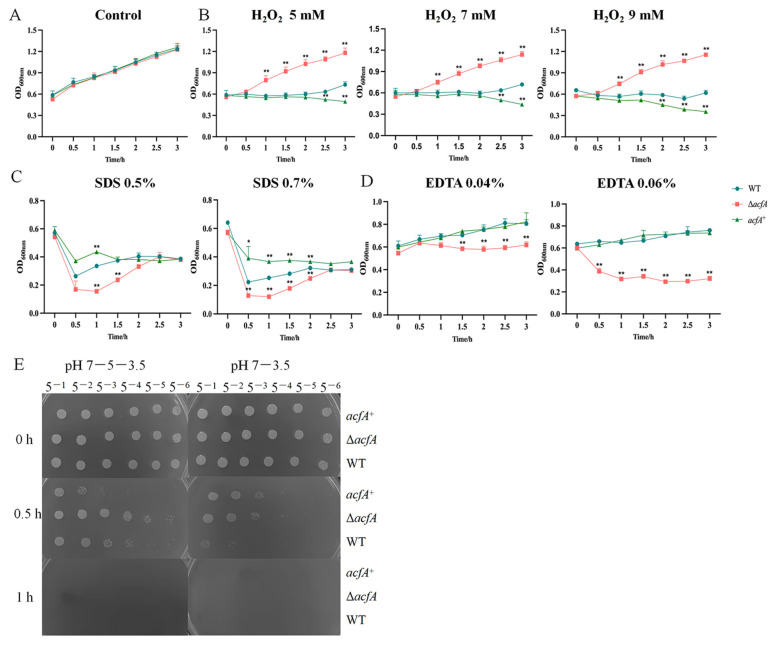
The role of AcfA in the stress response of *Vibrio parahaemolyticus*. All strains were initially adjusted to the same OD_600_, followed by stress testing under different environmental conditions. The experimental conditions were as follows: (**A**) LB broth; (**B**) LB broth supplemented with 5 mM, 7 mM, or 9 mM H_2_O_2_; (**C**) LB broth supplemented with 0.5% or 0.7% SDS; (**D**) LB broth supplemented with 0.04% or 0.06% EDTA. (**E**) Acid sensitivity assay. The indicated strains were pre-cultured at pH 5.0 or pH 7.0 for 1 h and then exposed to a lethal acidic pH (3.5). Bacterial cultures subjected to acid stress for 0 h, 0.5 h, and 1 h were collected and then serially diluted (five-fold), and an aliquot of 2.5 μL was spotted onto LB agar plates. The pictures shown are representative of three independent biological replicates. (* *p* ≤ 0.05, ** *p* ≤ 0.01).

Bacteria encounter diverse external environmental alterations. Pathogens that can cause successful infection in the host must adapt themselves to and thrive under variant environmental stresses. The roles of AcfA in the stress response of *V. parahaemolyticus* were defined. There is no significant difference in the growth of the three strains in LB broth with no addition of stressors (Figure 2A). For the salt stress assay, overnight-cultured strains were spotted onto LB plates containing 0.5%, 1%, 3%, 5%, or 7% (*w*/*v*) NaCl. No obvious growth variations were observed among these strains (Figure S2). For the H_2_O_2_ stress survival assay, we observed significant differences in bacterial growth in LB broth with various levels of H_2_O_2_ (Figure 2B); the Δ*acfA* strain grew obviously better than f both the wild-type and *acfA*^+^ strains. After being stressed with H_2_O_2_ (5 mM, 7 mM, 9 mM) for 1 h, the OD_600_ values of the Δ*acfA* strain decreased significantly compared to those of the wild-type strain. The *acfA*^+^ strain displayed no obvious alteration compared with the wild-type strain. For the SDS stress assay, all three strains displayed reduced growth in LB containing 0.5% (*w*/*v*) or 0.7% (*w*/*v*) SDS compared the control group with no SDS addition. The growth of the Δ*acfA* strain decreased significantly compared to that of the wild-type and *acfA*^+^ strains within 2 h after SDS stimulation. Yet, no obvious growth difference was observed among the strains under SDS stress for a longer period of time (Figure 2C). This suggests that AcfA is involved in the H_2_O_2_ stress response in *V. parahaemolyticus*.

After being stressed with 0.04% EDTA for 1.5 h, the OD_600_ values of the Δ*acfA* strain decreased significantly compared to those of wild-type and *acfA*^+^ strains. As the concentration of EDTA increased to 0.06%, the growth of the Δ*acfA* strain was obviously weakened throughout the detection period. Complementation with the *acfA* gene restored the growth of the Δ*acfA* strain (Figure 2D). This finding suggests that AcfA mediates EDTA resistance in *V. parahaemolyticus.*

Acid stress is a commonly encountered condition that affects bacterial survival. Here, the growth of the three bacterial strains under acid stress was determined. It can be seen that none of the strains could survive after being cultured at pH 3.5 for 1 h. When incubated at pH 7.0 for 1 h and then subjected to pH 3.5 for 0.5 h, all the strains could survive. When incubated at pH values from 5.0 (1 h) to 3.5 (0.5 h), the Δ*acfA* strain grew better than the wild-type and *acfA*^+^ strains. It is noticed that the *acfA*^+^ strain exhibited a prominent impairment in acid resistance and could not even survive at a dilution of 5^−5^ (Figure 2E). This finding indicates that the *acfA* gene is involved in the acid stress response in *V. parahaemolyticus.*

### 3.3. General Features of the Transcriptome Profile

**Figure 3:**
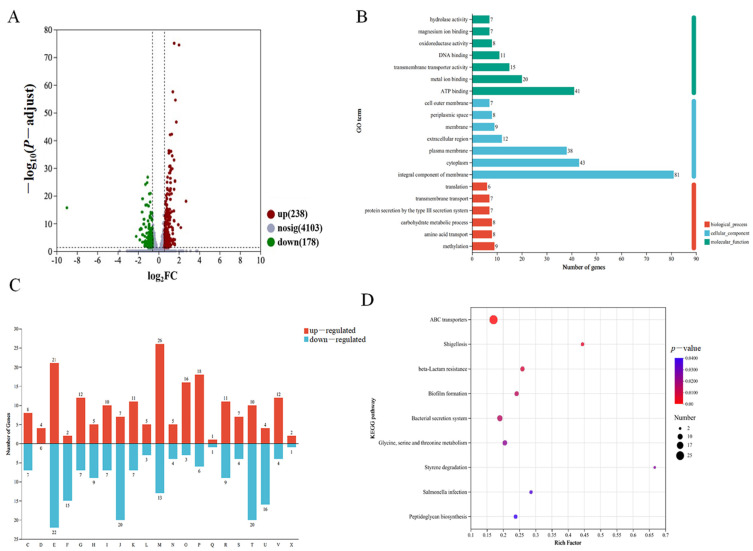
RNA-Seq analyses of the transcriptome alterations caused by the deletion of *acfA* in *V. parahaemolyticus*. (**A**) Volcano map showing the differentially expressed genes (DEGs) in the Δ*acfA* vs. WT strains. (**B**) GO functional categories of the DEGs. The number on each bar represents the number of DEGs in each term. (**C**) COG functional annotations of the DEGs. The COG functional description is represented by capital letters. D: Cell cycle control, cell division, chromosome partitioning; M: Cell wall/membrane/envelope biogenesis; N: Cell motility; O: Posttranslational modification, protein turnover, chaperones; T: Signal transduction mechanisms; U: Intracellular trafficking, secretion, and vesicular transport; V: Defense mechanisms; J: Translation, ribosomal structure, and biogenesis; K: Transcription; L: Replication, recombination, and repair; C: Energy production and conversion; E: Amino acid transport and metabolism; F: Nucleotide transport and metabolism; G: Carbohydrate transport and metabolism; H: Coenzyme transport and metabolism; I: Lipid transport and metabolism; P: Inorganic ion transport and metabolism; Q: Secondary metabolites biosynthesis, transport, and catabolism; S: Function unknown. (**D**) KEGG pathway enrichment analysis of the DEGs. The x-axis indicates the significance of the level of enrichment, and the y-axis indicates the KEGG pathway.

In an effort to better understand the cellular function of AcfA, we performed transcriptome profiling of the Δ*acfA* mutant and compared it to the wild-type strain to gain insight into the global transcriptional alterations produced by *acfA* deletion in *V. parahaemolyticus*. Four parallel Δ*acfA* vs. WT groups were set up, and the RNA-Seq data after quality checking are shown in Table S2. We discovered that 416 genes were differently expressed, with 238 up-regulated and 178 down-regulated (*p*-adjust < 0.05 and absolute FC ≥ 1.5) (Figure 3A).

Using the Gene Ontology (GO) database, all the differentially expressed genes (DEGs) were categorized into three main functional categories: molecular function (MF), cellular component (CC), and biological process (BP). In terms of BP, ATP binding was the most common (9.85%, 41/416), followed by metal ion binding (4.81%, 20/416) and transmembrane transporter activity (3.61%, 15/416). The most concentrated DEGs in CC were found in the integral component of the membrane (19.47%, 81/416), followed by the cytoplasm (10.34%, 43/416) and the plasma membrane (9.13%, 38/416). In terms of MF, the top three were methylation (2.16%, 9/416), amino acid transport (1.92%, 8/416), and carbohydrate metabolic process (1.92%, 8/416) (Figure 3B).

To provide a more intuitive explanation of the types and functions of genes with significant transcriptional changes, COG functional annotation analysis revealed that genes associated with cell wall/membrane/envelope biogenesis (26 DEGs), amino acid transport (21 DEGs), and inorganic ion transport and metabolism (18 DEGs) were significantly up-regulated in the Δ*acfA* strain compared with the wild-type strain. Furthermore, there were more down-regulated genes than up-regulated genes for nucleotide transport and metabolism, coenzyme transport and metabolism, translation, ribosomal structure and biogenesis, signal transduction mechanisms, intracellular trafficking, secretion, and vesicular transport (Figure 3C).

Based on the KEGG pathway analysis, the DEGs were divided into cellular processes (*n* = 25), human diseases (*n* = 23), genetic information processing (*n* = 11), environmental information processing (*n* = 46), organismal system (*n* = 6), and metabolism (*n* = 116). The top three pathways were membrane transport (8.65%, 36/416), amino acid metabolism (6.49%, 27/416), and carbohydrate metabolism (5.04%, 21/416) (Figure S3). The KEGG enrichment analysis indicated that the DEGs were mainly involved in nine pathways, including ABC transporters, bacterial secretion systems, biofilm formation, glycine, serine, and threonine metabolism, beta-lactam resistance, peptidoglycan biosynthesis, and so on (Figure 3D).

### 3.4. Validation of the Transcriptome Data by qRT-PCR

**Figure 4:**
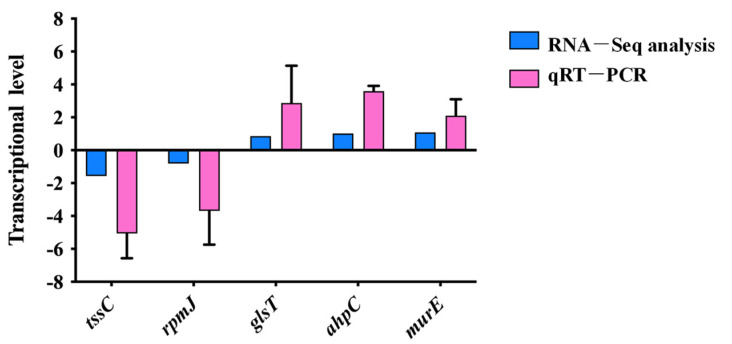
qRT-PCR validation of the RNA-Seq data. The relative expression of five differentially expressed genes detected by RNA-Seq was verified by qRT-PCR. The gene expression levels were normalized to the 16S rRNA level. Error bars indicate standard deviations. The blue columns indicate the results of RNA-Seq, while the red columns indicate the results from the qRT-PCR assay.

The transcriptome data were validated using qRT-PCR. Two significantly decreased genes and three significantly increased genes were chosen as target genes. The transcriptome data were found to be reliable, as evidenced by the constant pattern between the qRT-PCR findings of all examined genes and those of the transcriptome data, as seen in Figure 4.

### 3.5. AcfA Supports the Type III Secretion System, the Type VI Secretion System, and Biofilm Formation in V. parahaemolyticus

**Figure 5:**
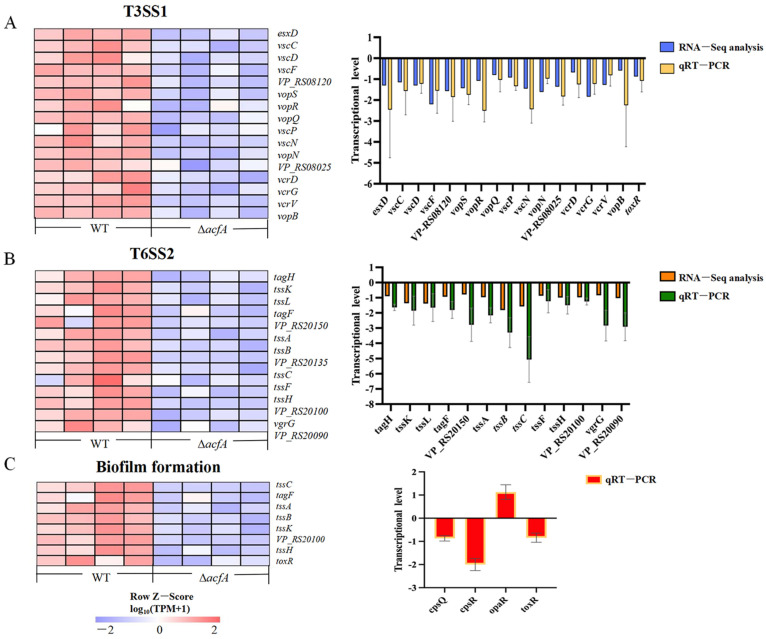
Heat map (left) and qRT-PCR verification (right) of the main differentially expressed genes. The expression levels of the genes in each sample were normalized by taking log_10_ (TPM+1), followed by Z-score normalization. (**A**) Differentially expressed genes (DEGs) involved in T3SS1. (**B**) DEGs involved in T6SS2. (**C**) DEGs involved in biofilm formation. In the heatmap, The red color denotes higher transcription and blue color denotes lower transcription.

The Type III secretion system (T3SS) and the Type VI secretion system (T6SS) are the two pivotal toxin-delivery systems used by Gram-negative bacteria. In *V. parahaemolyticus*, there are two T3SSs (T3SS1 and T3SS2) and two T6SSs (T6SS1 and T6SS2) that are encoded on the two different chromosomes. T3SS2 is primarily responsible for *V. parahaemolyticus*’s enterotoxicity and acute gastroenteritis, whereas T3SS1 plays important roles in adherence and cytotoxic activity against a variety of cell types [17,18,19,20]. Furthermore, T6SS1 is necessary for *V. parahaemolyticus* to be environmentally fit due to its antibacterial activity, whereas T6SS2 is involved in cellular adhesion [21,22,23]. The RNA-Seq results demonstrated that deletion of the *acfA* gene induced decreased transcriptional levels of both the T3SS1 and T6SS2 gene clusters (Figure 5A,B). A total of 16 genes encoding T3SS1 were significantly down-regulated in the Δ*acfA* mutant compared to those in the wild-type strain (*p*-adjust < 0.05). For instance, *vscF* (*VP_RS08155*) and *vcrG* (*VP_RS07995*), encoding the T3SS1 needle filament protein VscF and the T3SS1 chaperone VcrG, were down-regulated by 2.20- and 1.84-fold (log_2_FC), respectively. Transcription of *vopN* (*VP_RS08030*) and *vscN* (*VP_RS08035*), encoding for the two T3SS gatekeeper subunits, were down-regulated 1.61- and 1.45-fold (log_2_FC) in the Δ*acfA* mutant, respectively. In addition, 14 annotated T6SS2 genes displayed significantly lower expression levels in the Δ*acfA* strain relative to the wild-type strain (*p*-adjust < 0.05). For instance, *tssC* (*VP_RS20130*), *tssC* (*VP_RS20135*), and *tssB* (*VP_RS20140*), encoding components of the T6SS2 contractile sheath, were down-regulated by 1.10-, 1.57-, and 1.82-fold (log_2_FC), respectively (Table S3). To verify the effect of AcfA on the T3SS1 and T6SS2 systems, the transcriptional levels of the DEGs (16 involved in T3SS1 and 14 involved in T6SS2) in the Δ*acfA* strain vs. the WT strain were detected by qRT-PCR. The result demonstrated that deletion of *acfA* resulted in a reduction in the transcriptional levels of all the tested genes, in agreement with the RNA-Seq data (Figure 5A,B and Table S3).

In the absence of *acfA*, biofilm formation decreased significantly in the mutant strain compared to the wild-type strain, while biofilm formation was restored in the complemented strain (Figure 1C). The RNA-Seq data indicated that the biofilm formation pathway was significantly enriched (Figure 3D). Eight genes involved in biofilm formation were obviously down-regulated (Figure 5C). For instance, the transcriptional level of *toxR*, the positive regulator of biofilm formation in *V. parahaemolyticus*, was down-regulated (log_2_FC = 0.54) in the mutant strain (Table S3). Moreover, the transcriptional levels of three genes (*cpsQ*, *cpsR*, and *opaR*) closely related to biofilm formation in *V. parahaemolyticus* were determined, and the results are shown in Figure 5C. OpaR, the master quorum sensing regulator at high cell density, represses biofilm formation in *V. parahaemolyticus* [24]. The qRT-PCR data indicated that deletion of *acfA* resulted in a higher transcriptional level of *opaR* compared to the wild-type strain. CpsQ, a primary driver of biofilm formation, promotes biofilm formation by *V. parahaemolyticus* [25]. CpsR directly activates the transcription of *cpsQ* [26]. As shown in Figure 5C, the levels of both *cpsQ* and *cpsR* decreased in the mutant strain.

### 3.6. Deletion of acfA Caused Disorder of the Cell Envelope

**Figure 6:**
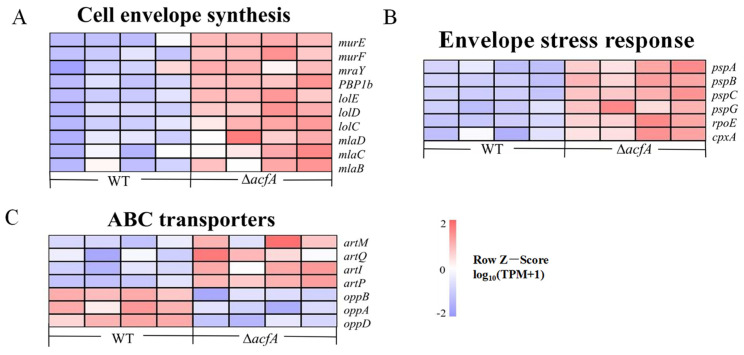
Heat map of the main differentially expressed genes involved in the envelope stress response, cell envelope synthesis, and ABC transporters. The expression levels of the genes in each sample were normalized by taking log_10_ (TPM+1), followed by Z-score normalization. The red color denotes higher transcription and blue color denotes lower transcription. (**A**) DEGs involved in cell envelope biosynthesis. (**B**) DEGs involved in the cell envelope stress responses. (**C**) DEGs involved in ABC transporters.

The cell envelope is a multilayered structure that separates the cytoplasm of bacterial cells from ever-changing outside conditions. It consists of the inner membrane (IM), the periplasm, and the outer membrane (OM), an asymmetric bilayer with phospholipids in the inner leaflet and lipopolysaccharides in the outer leaflet. The cell envelope is crucial for regulating bacterial pathogenicity, division, and shape, as well as resistance to external stimuli [27]. In this study, 10 DEGs involved in cell envelope biosynthesis were significantly up-regulated in the Δ*acfA* mutant compared to the wild-type strain (*p*-adjust < 0.05). For instance, expression levels of the *murE*, *murF*, and *mraY* genes involved in peptidoglycan synthesis were up-regulated 1.09-, 0.94-, and 0.91-fold (log_2_FC) in the Δ*acfA* strain vs. the wild-type strain. MurE and MurF add mDAP and D-Ala-D-Ala to the UM-tripeptide, respectively [28]. The transcriptional levels of another three genes, *lolC*, *lolD*, and *lolE*, encoding for the lipoprotein-releasing ABC transporter, were also elevated significantly (0.99-, 1.16-, and 1.01-fold) in the Δ*acfA* strain compared to the wild-type strain. Most lipoproteins in Gram-negative bacteria are transported to the OM via the LolABCDE route. Furthermore, deletion of *acfA* caused a significant elevation in the expression of three genes involved in the maintenance of the OM lipid asymmetry (Mla) system, *mlaB*, *mlaC*, and *mlaD* (0.78-, 0.60-, and 0.62-fold (log_2_FC), respectively) (Figure 6A and Table S3).

### 3.7. Deletion of acfA Caused Enhancement of the Envelope Stress Response System in V. parahaemolyticus

Bacteria have developed diverse envelope stress response systems in order to survive in the changing circumstances faced both in the environment and within the host, such as the σ^E^ response, two-component systems, the phage shock protein (Psp) response, the Rcs phosphorelay system, and outer membrane vesicle formation. In *V. parahaemolyticus*, deletion of the *acfA* gene induced a significant increase in the transcriptional levels of genes involved in three envelope stress response systems: the σ^E^ response, the CpxRA system, and the Psp response. The expression levels of *rpoE*, *cpxA*, *pspA*, *pspB*, *pspC*, and *pspG* were up-regulated 0.87-, 1.04-, 0.63-, 0.82-, 0.71-, and 0.80-fold (log_2_FC), respectively (Figure 6B and Table S3).

### 3.8. AcfA Had an Impact on the Arginine Transport System in V. parahaemolyticus

In addition to being a ubiquitous amino acid needed for protein synthesis, arginine is also crucial for several other facets of cellular development and function. It serves as a substrate for polyamine production and is indispensable for acid resistance in *Escherichia coli* [29]. In the Δ*acfA* vs. wild-type comparison, the *artPIQM* gene cluster, encoding the ATP-binding cassette-type (ABC) transport system for the import of arginine was up-regulated significantly (*p*-adjust < 0.05). Transcription of *artP*, *artI*, *artQ*, and *artM* was up-regulated 1.29-, 1.54-, 0.87-, and 1.42-fold (log_2_FC), respectively (Figure 6C and Table S3).

### 3.9. Deletion of acfA Led to Impairment of the Oligopeptide Permease System in V. parahaemolyticus

The oligopeptide permease (Opp) system is responsible for peptide uptake in bacteria. It is composed of five members, which are encoded by the *oppABCDF* operon. OppA, a membrane-associated lipoprotein, binds with peptides in the external environment and delivers them to the two channel-forming transmembrane proteins, OppB and OppC, through which the peptide is translocated into the cytoplasm with the aid of the two membrane-bound cytoplasmic ATP-binding proteins, OppD and OppF, for ATP supply. The RNA-Seq results demonstrated that, when *acfA* expression was disrupted, *oppB* (0.76-fold), *oppC* (0.90-fold), and *oppD* (0.79-fold) (log_2_FC) were obviously down-regulated in contrast with the wild-type strain (Figure 6C and Table S3). In *V. alginolyticus*, OppABCDF was demonstrated to play a key role in bacterial pathogenesis at multiple steps, including adhesion, biofilm production, and hemolytic activity [30].

## 4. Discussion

Most pathogenic bacteria need to be able to live both within and outside of their host. Attachment to and colonization of abiotic or biotic surfaces is a typical way for microbes to improve their capacity to survive under distinct conditions. As an aquatic inhabitant, *Vibrio parahaemolyticus* is often found attached to the chitinous exoskeletons of zooplankton. Attachment colonization factors (ACFs) have been shown to be essential for *Vibrio* in host colonization. AcfA, as one of the ACFs, plays a critical role in effective colonization by *V. cholerae* of the mouse intestine. In this work, we constructed a markerless in-frame deletion mutant strain, Δ*acfA*, and a complemented strain, *acfA*^+^, and elucidated the global roles of AcfA in *V. parahaemolyticus* by RNA-Seq assay.

Herein, an in-frame deletion mutant strain, Δ*acfA*, lacking nucleotides 4 to 645 of the *acfA* gene, and the complementary strain *acfA*^+^ of *V. parahaemolyticus* were successfully generated. Deletion of *acfA* had no impact on bacterial growth in nutrient-rich medium but caused a significant reduction in virulence-related phenotypes, including mucus adhesion, biofilm formation, hemolytic activity, and the accumulated mortality of zebrafish. The RNA-Seq results showed that deletion of *acfA* caused a significant decrease in the transcription of 16 genes involved in T3SS1 and 14 genes involved in T6SS2. It takes both T3SS1 and T3SS2 for *V. parahaemolyticus* to reach its full virulence. T3SS2 is primarily responsible for *V. parahaemolyticus*’s enterotoxicity and acute gastroenteritis, whereas T3SS1 plays important roles in adherence and cytotoxic activity against a variety of cell types [17,18,19,20]. Furthermore, T6SS1 is necessary for *V. parahaemolyticus* to be environmentally fit due to its antibacterial activity, whereas T6SS2 is involved in cellular adhesion [21,22,23]. Oligopeptide permeases (Opp), ATP-binding cassette transporters, capture and deliver extracellular peptides to the cytoplasm for carbon and nitrogen supply. Of note, Opp systems are also involved in bacterial virulence. In *V. alginolyticus*, OppABCDF contributes to multiple steps of pathogenesis, including adhesion, biofilm production, hemolytic activity, and host infection [30]. Furthermore, deletion of *oppA* induces a great reduction in cell adherence and mouse infectivity in *Streptococcus suis* serotype 2 [31]. Disruption of *acfA* expression caused a significant reduction in the expression of genes encoding Opp systems, including *oppB*, *oppC*, and *oppD*. It can be concluded that deletion of the *acfA* gene resulted in deficiency of the T3SS1, T6SS2, and also Opp systems and thus reduced the virulence of *V. parahaemolyticus*.

Furthermore, AcfA was demonstrated to be involved in the stress adaptation of *V. parahaemolyticus* in this work. The Δ*acfA* strain displayed a significant growth deficiency compared to the wild-type strain under EDTA stress, while in the absence of *acfA*, the bacteria exhibited better growth under high salt (7% NaCl), H_2_O_2_ (0.5 mM), and acid stress. The cell envelope is a complex, extracytoplasmic compartment insulating the cytoplasmic space from an often chaotic outside world. The Gram-negative bacterial envelope comprises two structurally distinct lipid bilayers—the inner membrane (IM) and the outer membrane (OM)—with a thin periplasm and peptidoglycan (PG) cell wall in the space between them [32]. PG is produced in multiple stages occurring in three distinct cell compartments: the periplasm, membrane, and cytoplasm. UDP-N-acetylglucosamine is stepwise catalyzed by MurA and MurB to form the main sugar backbone, UDP-N-acetylmuramic acid (UM), to which L-alanine and D-glutamate are added through the actions of MurC and MurD, respectively [33]. Afterward, meso-diaminopimelate (DAP) is ligated to the growing chain by MurE. Finally, a D-alanyl-D-alanine dipeptide is connected to the UM-tripeptide via catalysis by MurF to form the UM-pentapeptide, which is subsequently combined with a membrane-bound lipid carrier, undecaprenol-phosphate, by MraY, generating Lipid I [34]. MurG then associates a GlcNAc group with this molecule, transforming it into Lipid II, which is flipped toward the periplasmic space and cross-linked to form PG under the actions of PBPs, Lpo regulators, and SEDS (shape, elongation, division, and sporulation) family proteins [35,36]. Here, we discovered that deletion of *acfA* induced the elevated expression of multiple genes annotated as participating in PG biosynthesis in the Δ*acfA* strain vs. wild-type strain, such as *murE* (1.09-fold), *murF* (0.94-fold), *mraY* (0.91-fold), and *PBP1b* (0.90-fold) (Log_2_FC), indicating a key role for AcfA in PG production. Furthermore, genes involved in maintenance of the lipid asymmetry (Mla) route, including *mlaB* (0.78-fold), *mlaC* (0.60-fold), and *mlaD* (0.62-fold) (Log_2_FC), were also up-regulated in the Δ*acfA* mutant. The Gram-negative bacterial OM has an asymmetric lipid architecture, with lipopolysaccharides in the outer leaflet and phospholipids (PLs) in the inner leaflet, protecting the cell from the detrimental external environment. The Mla route, perhaps the best-understood PL transporter, is thought to be critical for upholding OM asymmetry and integrity by transporting PLs between the IM and OM [37,38]. Moreover, the absence of AcfA led to significantly higher expression levels of members of the lipoprotein transport system (Lol), including *lolC* (0.99-fold), *lolD* (1.16-fold), and *lolE* (1.01-fold) (Log_2_FC). The Lol system, making the major contribution to the trafficking of lipoproteins to the OM of G^-^ bacteria, consists of an ABC transporter, LolCDE, the periplasmic chaperone LolA, and the OM-anchored lipoprotein LolB. LolCDE delivers matured lipoproteins translocated onto the outer leaflet of IM to LolA, which then shuttles lipoproteins across the periplasm to LolB. Finally, the lipoproteins are inserted into the inner leaflet of the OM, providing a covalent link between the OM and peptidoglycan. The Lol route is critical for OM integrity and bacterial pathogenesis [39].

Bacteria have developed distinct envelope stress response systems in order to adapt to a wide range of environmental and mammalian host circumstances, including the σ^E^ response, the CpxAR and BaeSR two-component systems, the phage shock protein (Psp) response, the Rcs phosphorelay system, and outer membrane vesicle formation. Each stress response detects a distinct activation signal that indicates a certain form of extracytoplasmic disruption [40]. Of note, the σ^E^ response, the CpxRA system, and the Psp response were all enhanced in the Δ*acfA* mutant of *V. parahaemolyticus*: *rpoE* (0.87-fold), *cpxA* (1.04-fold), *pspA* (0.63-fold), *pspB* (0.82-fold), *pspC* (0.71-fold), and *pspG* (0.80-fold) (Log_2_FC). σ^E^, an extracytoplasmic function σ factor, is a subunit of the RNA polymerase (RNAP) holoenzyme and initiates transcription of numerous target genes under the indicated conditions. The σ^E^ system responds to a wide range of stresses, including misfolded envelope proteins and alterations in lipopolysaccharide production. In *V. parahaemolyticus*, the σ^E^ system was indicated to be involved in polymyxin B, ethanol, and high-temperature stresses, as well as intestinal colonization. Moreover, OmpU is not essential for RpoE function in *V. parahaemolyticus* [41]. In *V. cholerae*, RpoE is required for bacterial growth under 3% ethanol stress, and OmpU serves to trigger RpoE function [42,43]. The Cpx (conjugative plasmid expression) response system is a two-component system, consisting of CpxA (a sensor kinase) and CpxR (a response regulator). A great number of studies on the function and regulation mechanism of CpxRA have been conducted in *Escherichia coli*, and the Cpx response system can be induced and activated by multiple extracellular cues, including PG homeostasis [44], lipoprotein trafficking perturbation [45], adhesion [46], impaired efflux of the siderophore enterobactin [47], and other stresses. The Cpx response system was also found in *Vibrionaceae*. In *V. cholerae*, activation of the Cpx pathway is caused by iron chelation, toxic chemicals, or loss of certain RND efflux components. The signals inducing the Cpx response system are different from those in *E. coli*. For instance, Cpx activation occurs with increasing osmolarity in *E. coli*, but with salinity rather than osmolarity in *V. cholerae* [48,49]. This work indicated that deletion of the outer membrane beta-barrel protein AcfA activates the Cpx pathway in *V. parahaemolyticus*. The Psp (phage shock protein) stress response is mainly activated by the dissipation of the proton motive force (PMF) caused by inner membrane perturbations. It has been found that overexpression of the type II secretion system can induce the Psp response in *V. cholerae*. Furthermore, stationary-phase growth also increases the Psp response [50]. Though it was previously believed that variations in the PMF directly cause the Psp response, there may be more inducing signals. It can be seen that the absence of AcfA, an outer membrane beta-barrel protein, induced disorder of cell envelope biosynthesis, such as peptidoglycan production, maintenance of lipid asymmetry, and lipoprotein translocation, and in turn activated the cell envelope stress response pathway, including the σ^E^ response, the CpxRA system, and the Psp response, to rescue the dysfunction.

**Figure 7:**
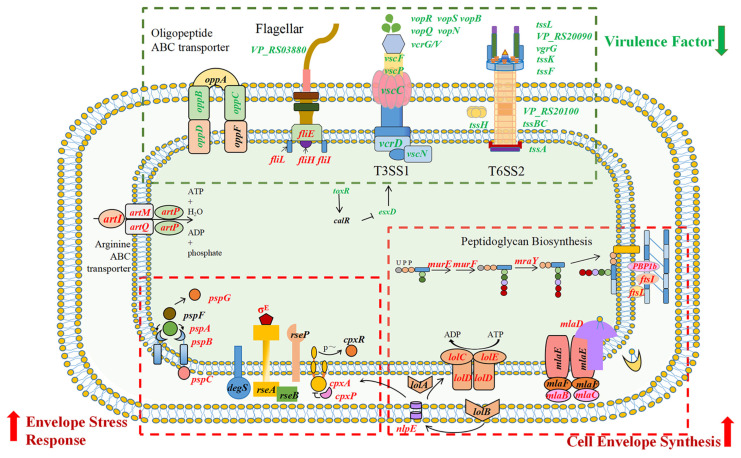
Overall regulatory functions of AcfA in *V. parahaemolyticus*. The three boxes indicate the major pathways regulated by AcfA. Red and green indicate up- and down-regulated genes, respectively.

In conclusion, we present the genome-wide transcriptional alterations that occur in the absence of *acfA* and report that AcfA plays an important role in potential virulence and cell envelope stress response regulation in *V. parahaemolyticus* (Figure 7). Our findings shed light on AcfA’s functions and its underlying mechanism in *V. parahaemolyticus*.

## Figures and Tables

**Table 1:** Strains and plasmids used in this study.

Strain or Plasmid	Relevant Characteristics	Reference or Source
*Escherichia coli*		
DH5α λpir	Host for π-requiring plasmids	Lab collection
SM10 λpir	Host for π-requiring plasmids, conjugal donor	Lab collection
*Vibrio parahaemolyticus*		
RIMD2210633	Clinical isolate, Amp^r^	Lab collection
∆*acfA*	RIMD2210633, in-frame deletion in *acfA*,Amp^r^	This study
*acfA^+^*	∆*acfA* carrying the complementary plasmid, pMMB207-*acfA*, Amp^r^, Cm^r^	This study
Plasmids		
pDM4	Suicide vector, pir dependent, R6K, SacBR, Cm^r^	[11]
pMMB207	IncQ lacI^q^ Δ*bla* P_tac-lac_lacZa, Cm^r^	[12]
pDM4-∆*acfA*	pDM4 with the *acfA* fragment deleted 4th to 645th nucleotides, Cm^r^	This study
pMMB207-*acfA*	pMMB207 with the intact *acfA* gene	This study

Note: Amp^r^ represents ampicillin-resistant, Cm^r^ represents chloramphenicol-resistant.

**Table 2:** Primers used for strain construction.

Primers	Sequence(5′-3′)
*acfA*-up-F	GAGCGGATAACAATTTGTGGAATCCCGGGATAACTCGGACGGAGACCA
*acfA*-up-R	CCTTAAATTACATTCTAATTTTGCCTCTTCAAATC
*acfA*-down-F	AATTAGAATGTAATTTAAGGAAACGATAATGAAAA
*acfA*-down-R	GCGGAGTGTATATCAAGCTTATCGATACCAACCGTATCATTCCCGTA
*acfA*-out-F	TTTCGGGTGGTGGCGTGGAC
*acfA*-out-R	TCCGAATCCTCTACCACTTA
*acfA*-in-F	CCTGCTCAGTTTCGGTAT
*acfA*-in-R	CCTTGGCGTCGTATTGTG
*acfA*-com-F	TCGGTACCCGGGGATCCTCTAGTAAGGAGGTAGGATAATAATGAACAAAACACTTCTTGCGTTAC
*acfA*-com-R	TCCGCCAAAACAGCCAAGCTTTAGTGATGATGATGATGATGAAAGTAGTAAGTTGTGCCTACG

**Table 3:** Bacterial adherence to mucus.

Bacterial Strain	Number of Bacteria (Cells)
WT	1101
Δ*acfA*	174
*acfA* ^+^	1443

## Data Availability

The data in this study are included in this published article and its Supplementary Information Files. The transcriptome data have been uploaded to NCBI (PRJNA1071496).

## Supplementary Materials

The following supporting information can be downloaded at: https://www.mdpi.com/article/10.3390/microorganisms13010007/s1, Figure S1: Verification of the Δ*acfA* mutant strain and the complementary strain *acfA*^+^; Figure S2: The roles of AcfA in the salt stress response of *V. parahaemolyticus*; Figure S3: KEGG annotation of the DEGs in of the Δ*acfA* strain vs. the WT strain; Table S1: qRT-PCR primers; Table S2: RNA-seq data after quality checking; Table S3: Information about the differentially expressed genes in Δ*acfA* vs. WT.
