# Supplementary material for: AcfA Regulates the Virulence and Cell Envelope Stress Response of Vibrio parahaemolyticus
Source: Microorganisms. 2024 Dec 24;13(1):7. doi: 10.3390/microorganisms13010007 (PMC11767970; doi:10.3390/microorganisms13010007)
Supplement: Supplementary file 1 [file microorganisms-13-00007-s001.zip › microorganisms-3327906-supplementary.pdf]

microorganisms

MDPI

Supplementary Materials

1. Supplementary Figures and Tables

1.1 Supplementary Figures

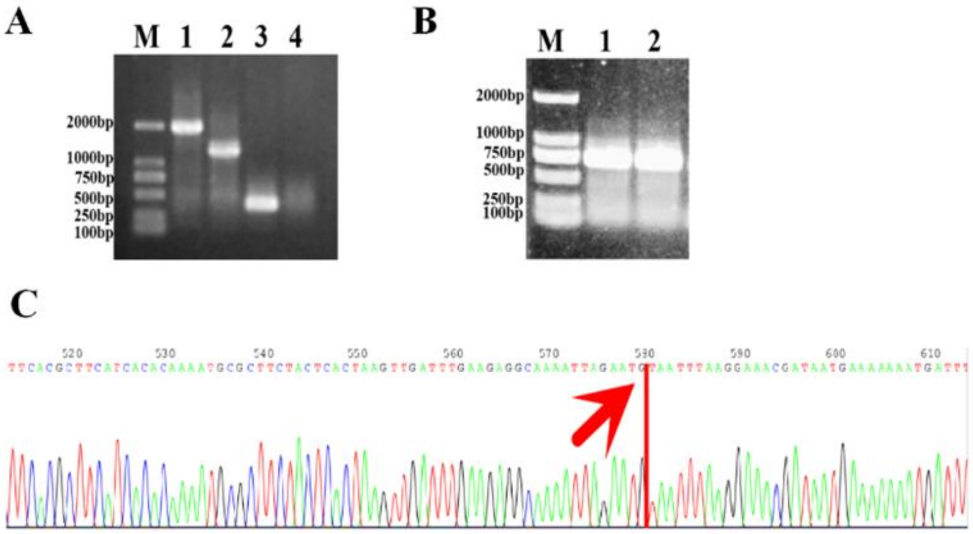

Supplementary Figure S1. Verification of the Δ*acfA* mutant strains and the complementary strain *acfA*$^+$. **(A)** Verification of the Δ*acfA* mutant by PCR. M, DNA marker 2000; Lane 1, band generated by PCR with the primers of acfA-out-F and acfA-out-R in WT; Lane 2, band generated by PCR with the primers of acfA-out-F and acfA-out-R in Δ*acfA*; Lane 3, band generated by PCR with the primers of acfA-in-F and acfA-in-R in WT; Lane 4, band generated by PCR with the primers of acfA-in-F and acfA-in-R in Δ*acfA*. **(B)** Verification of *acfA*$^+$ by PCR. M, DNA marker 2000; Lane 1, band generated by PCR with the primers of acfA-com-F and acfA-com-R in WT; 2, band generated by PCR with the primers of acfA-com-F and acfA-com-R in *acfA*$^+$. **(C)** Sequencing result of the Δ*acfA* mutant and the arrow indidated the deletion region of *acfA* gene.

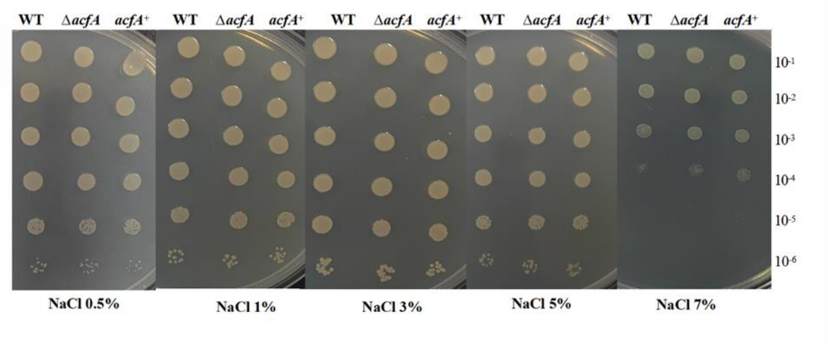

Supplementary Figure S2. The roles of AcfA in the salt stress response of *V. parahaemolyticus*. All strains were first diluted to OD_{600} = 1 and then serial dilutions were performed as indicated, and each dilution was spotted on LB agar plates with 0.5%, 1%, 3%, 5%, and 7% (w/v) and then incubated at 37 ^{o}C.

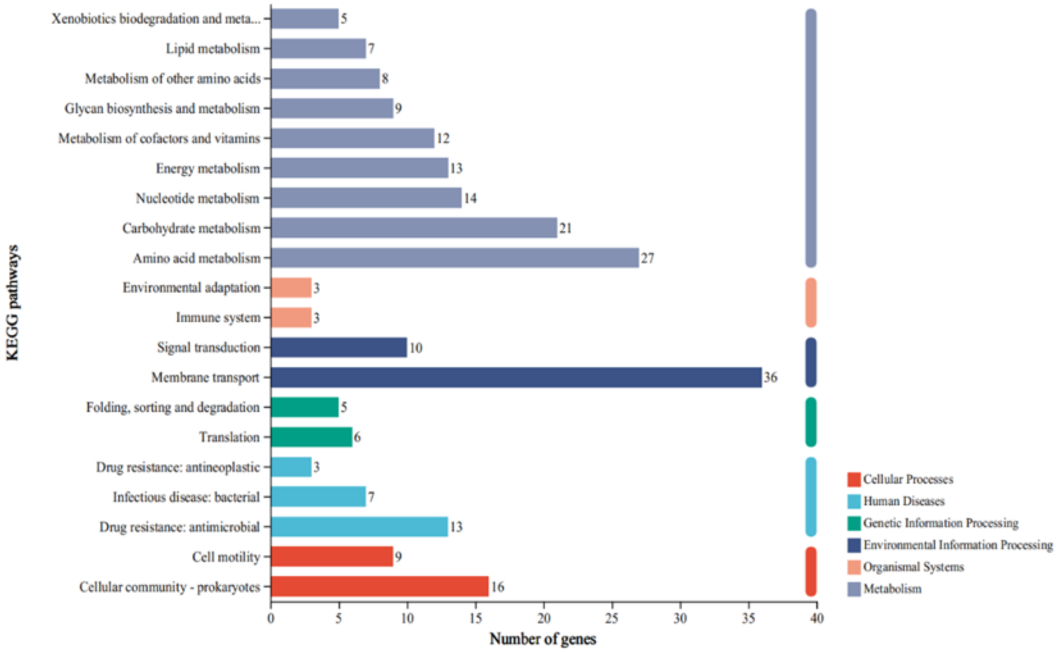

Supplementary Figure S3. KEGG annotation of the DEGs in the comparison of Δ*acfA* *vs* WT.

1.2 Supplementary Tables

Table S1 qRT-PCR primers

Primers	Sequence(5'-3')
esxD-F	TCGTTGAAGTGTAGTCCGCC
esxD-R	AACGAGCAGCAGTCACAAGT
vscC-F	GCAGTTTTGCCACGAGGTTT
vscC-R	TTGCTTCGGTATTGAGCCGT
vscD-F	GTGCCTTTGCTGACTTTGGG
vscD-R	AAACCACACGCTTTTACGGC
vscF-F	AACAAGCGAAAGACGCGAAC
vscF-R	GAGTGGTGGTTGCGTTGATG
VP_RS08120-F	AGTGATTGCCGTGTCTTGGT
VP_RS08120-R	GGATTTTGCTCACCGTTGCC
vopS-F	TTACCGAGGTCTTTGTCCGC
vopS-R	ATTGGTGGGGAAGGCATGTT
vopR-F	ACATAAAGGTGCGTGCCAGA
vopR-R	ATGTCGGGCACACCAGAAAA
vopQ-F	TTCGCCGTAGATTCCGTCTG
vopQ-R	GCTCAAGCCCGAATGAACAC
vscP-F	CCCCCACAACCTACTCAACC
vscP-R	TCTCATTCGATGCCGTTGGT
vscN-F	TCCCGTTTATCGTGATGCCC
vscN-R	TCTATGGAGCGAACGCCAAG
vopN-F	GCCAATCCGGAAAGCAAAC
vopN-R	GGTGTCGATTTGCGACCAA
VP_RS08025-F	GTCCCACGCTTCCTCTTCTT
VP_RS08025-R	TGCTGACGACGAACAGAGAC
vcrD-F	GCAACACGACGGCAATACTC
vcrD-R	AAGGTAGGGCAACGCAAAGA
vcrG-F	ACGCCGAGCAAGAACTCATC

vcrG-R	CTAGATCACCATACCGCGCA
vcrV-F	GCAATATCCCCAGTCGCTGA
vcrV-R	CAGCTTGGAACCGAAAGCAC
vopB-F	ATCGACATTCACACCCGCTT
vopB-R	TTATGATCGCTGGTGGCGTC
tagH-F	TGGTTGACGCGATTGGTTTG
tagH-R	GCCAAAATCGAGAGCAGTGG
tssK-F	CCCCACGTATATGAGCCACC
tssK-R	TTCTGCGTCCCCAGCATTTT
tssL-F	CAAAGGTCTCGCATCACCCA
tssL-R	CGGGCAACTTCTCTTTCCGA
tagF-F	CGTTCCAAGTGTCACCCAGT
tagF-R	CGCCTAAGTGGGGCTACATC
VP_RS20150-F	GGATGGGACTCTGCGTCTTC
VP_RS20150-R	CACTGCCTATGGGTTGGTGA
tssA-F	GCCAACCAAACGGAAGTAGC
tssA-R	CGAGCCTCACTCACCAGTTT
tssB-F	GCAAAGTCGCCCATAACACC
tssB-R	GTACGGAAGCCTCGTGTTCA
tssC-F	TGAAGGGGACGGTTTCTTGG
tssC-R	CCACGCCATGAGCCTTCTAA
tssF-F	TTGGTAAGCTGCCATCGTGT
tssF-R	CGGGCTACCAAAGGTTCAGT
tssH-F	CTCAATGCAACACGCGAACA
tssH-R	GCTGCGGTACAACTTTCTGC
VP_RS20100-F	TTTCGCCCTGCTTAGTGATGT
VP_RS20100-R	AGCAATGGACCGTGAAGCAA
vgrG-F	CTGGAAGAGAATACGGGGGC
vgrG-R	TCGGGTAGGGCAAGAGGTTA
VP_RS20090-F	TTTGTCACCTTTTCTCGCGG
VP_RS20090-R	AACCAGCAGCAGTGATTGGA

cpsQ-F	ATGGCCCCTAATCCTCGACA
cpsQ-R	CTAGCGTCTTGGCCTCATGT
cpsR-F	TTGGAGTCGCACTCTGGTCAA
cpsR-R	TGCACGCGACACACCAAGTT
OpaR-F	CGCTCGTGAAAACATCGCAA
OpaR-R	ATCACGAGTTGATGCGCTCC
VP_RS21870-F	GTGGCGAGTGACCTTTCGTA
VP_RS21870-R	ATGATGATAGCGGTGCGTGT
toxR-F	AGGAAGCAACGAAAGCCGTA
toxR-R	CTCAAAACCTTGCTCACGCC
acfA-F	ACCTTGGCGTCGTATTGTGT
acfA-R	TGTCGTTGGCTCCAGTGTTT
rpmJ-F	ATGAAAGTTCGTGCTTCCGT
rpmJ-R	TTTGTGCTTTGGCTCACTGC
gltS-F	TCGCGGGTTCGATTACTCTG
gltS-R	AAACCAAAAGTGGCCGATGC
tcdA-F	TTGCGTGTTTTCAACCGAGC
tcdA-R	CATGCGTTTTGGACCTTCCG
murE-F	ATGGGCACCACAGGTAATGG
murE-R	GCCAAAGAAGCCAGCGTATG
ahpC-F	AGCTTGGCACGATAGCTCAG
aphC-R	CACGCATCACACCGAAGTTG
16sRNA-F	CTGGAACTGAGACACGGTCC
16sRNA-R	CTCGCACCCTCCGTATTACC

Table S2 RNA-seq data after quality checking

Sample	Clean Reads	Clean Bases(bp)	Clean Q20%	Clean Q30%	Clean Error Rate (%)
WT-1	27658766	3326033550	98.46	94.99	0.0241
WT-2	26431920	3343250308	98.37	94.78	0.0243
WT-3	26804372	3332865955	98.47	94.98	0.0241
WT-4	25162604	3168695525	98.47	95.00	0.0241
Δ*acfA*-1	27328730	3386045497	98.34	94.69	0.0244
Δ*acfA*-2	27358980	3400833728	98.29	94.57	0.0245
Δ*acfA*-3	28642854	3443965066	98.42	94.94	0.0242
Δ*acfA*-4	27085976	3384394616	98.29	94.60	0.0245

Clean reads: the number of bars with paired end reads after QC (quality control); Clean bases: the number of bars with paired end reads for QC multiplied by the length gives the number of total bases post QC; Clean Q20, Q30: the percentage of total bases with phred values greater than 20 and 30 were calculated after data quality control respectively. Clean error rate: Post QC base error rate.

Table S3 The information about the differentially expressed genes in Δ*acfA* *vs* WT

Gene ID	Gene name	Gene description	Log_2FC (Δ*acfA* / WT)	*p*-adjust
**Type III secretion system**				
VP_RS08180	*esxD*	type III secretion system regulon anti-activator ExsD	-1.30	8.57E-25
VP_RS08170	*vscC*	SctC family type III secretion system outer membrane ring subunit VscC	-1.15	1.55E-05
VP_RS08165	*vscD*	SctD family type III secretion system inner membrane ring subunit VscD	-1.30	0.000230837
VP_RS08155	*vscF*	type III secretion system needle filament protein VscF	-2.20	5.31E-06
VP_RS08120	*VP_RS08120*	CesT family type III secretion system chaperone	-1.57	1.37E-06
VP_RS08115	*vopS*	T3SS effector adenosine monophosphate-protein transferase VopS	-1.43	3.35E-12
VP_RS08105	*vopR*	type III secretion system effector VopR	-1.08	0.00256482
VP_RS08095	*vopQ*	type III secretion system effector VopQ	-0.80	4.73E-05
VP_RS08045	*vscP*	type III secretion system needle length determinant VscP	-0.92	0.030534767
VP_RS08035	*vscN*	SctN family type III secretion system ATPase VscN	-1.45	1.84E-10
VP_RS08030	*vopN*	SctW family type III secretion system gatekeeper subunit VopN	-1.61	8.44E-15
VP_RS08025	*VP_RS08025*	TyeA family type III secretion system gatekeeper subunit	-1.36	0.006672611
VP_RS08005	*vcrD*	SctV family type III secretion system export apparatus subunit VcrD	-0.68	0.012927068
VP_RS07995	*vcrG*	LcrG family type III secretion system chaperone VcrG	-1.84	0.000100088
VP_RS07990	*vcrV*	type III secretion system needle tip protein VcrV	-1.27	2.34E-09
VP_RS07980	*vopB*	type III secretion system translocon subunit VopB	-0.59	8.73E-05
**Type VI secretion system**				
VP_RS20180	*tagH*	type VI secretion system-associated FHA domain protein TagH	-0.90	9.13E-05
VP_RS20170	*tssK*	type VI secretion system baseplate subunit TssK	-1.37	4.56E-06
VP_RS20165	*tssL*	type VI secretion system protein TssL, long form	-1.39	0.000649015
VP_RS20155	*tagF*	type VI secretion system-associated protein TagF	-0.94	0.030957361
VP_RS20150	*VP_RS20150*	protein phosphatase 2C domain-containing protein	-0.79	0.027280761
VP_RS20145	*tssA*	type VI secretion system protein TssA	-0.97	7.42E-06

VP_RS20140	*tssB*	type VI secretion system contractile sheath small subunit	-1.82	1.90E-08
VP_RS20135	*tssC*	type VI secretion system contractile sheath large subunit	-1.57	1.34E-19
VP_RS20130	*tssC*	type VI secretion system contractile sheath large subunit	-1.10	0.006609211
VP_RS20115	*tssF*	type VI secretion system baseplate subunit TssF	-0.87	0.048479619
VP_RS20105	*tssH*	type VI secretion system ATPase TssH	-0.99	6.39E-06
VP_RS20100	*VP_RS20100*	type VI secretion system tube protein Hcp	-0.98	5.57E-17
VP_RS20095	*vgrG*	type VI secretion system tip protein VgrG	-0.84	1.69E-08
VP_RS20090	*VP_RS20090*	PAAR domain-containing protein	-1.03	0.00525715
**ABC transporters**				
VP_RS18270	*artM*	arginine ABC transporter permease ArtM	1.42	0.000202483
VP_RS18265	*artQ*	arginine ABC transporter permease ArtQ	0.87	0.006366003
VP_RS18260	*artI*	arginine ABC transporter substrate-binding protein	1.54	1.41E-11
VP_RS18255	*artP*	arginine ABC transporter ATP-binding protein ArtP	1.29	5.63E-15
VP_RS10165	*oppB*	oligopeptide ABC transporter permease OppB	-0.76	0.041265608
VP_RS10160	*oppC*	oligopeptide ABC transporter permease OppC	-0.90	0.001694508
VP_RS10155	*oppD*	ABC transporter ATP-binding protein	-0.79	9.98E-05
**Envelope stress response**				
VP_RS13390	*pspG*	envelope stress response protein PspG	0.80	5.63E-15
VP_RS05710	*pspC*	envelope stress response membrane protein PspC	0.71	1.26E-18
VP_RS05705	*pspB*	envelope stress response membrane protein PspB	0.82	1.93E-17
VP_RS05700	*pspA*	phage shock protein PspA	0.63	2.76E-13
VP_RS16805	*nlpE*	copper resistance protein NlpE	1.53	7.92E-76
VP_RS14065	*cpxA*	envelope stress sensor histidine kinase CpxA	1.04	1.69E-12
VP_RS14055	*cpxP*	CpxP family protein	1.21	9.36E-37
VP_RS12550	*rpoE*	RNA polymerase sigma factor RpoE	0.87	2.06E-21
**Cell envelope synthesis**				
VP_RS02180	*mraY*	phospho-N-acetylmuramoyl-pentapeptide-transferase	0.91	7.39E-05
VP_RS02175	*murF*	UDP-N-acetylmuramoyl-tripeptide-D-alanyl-D-alanine ligase	0.94	9.04E-11

VP_RS02170	*murE*	UDP-N-acetylmuramoyl-L-alanyl-D-glutamate-2,6-diaminopimelate ligase	1.09	6.41E-12
VP_RS12125	*PBP1b*	penicillin-binding protein 1B	0.90	2.97E-14
VP_RS04785	*lolE*	lipoprotein-releasing ABC transporter permease subunit LolE	1.01	1.14E-13
VP_RS04780	*lolD*	lipoprotein-releasing ABC transporter ATP-binding protein LolD	1.16	1.45E-14
VP_RS04775	*lolC*	lipoprotein-releasing ABC transporter permease subunit LolC	0.99	1.09E-14
VP_RS13060	*mlaD*	outer membrane lipid asymmetry maintenance protein MlaD	0.62	3.51E-06
VP_RS13055	*mlaC*	phospholipid-binding protein MlaC	0.60	6.59E-07
VP_RS13050	*mlaB*	lipid asymmetry maintenance protein MlaB	0.78	3.12E-07
**Other categories**				
VP_RS04000	*toxR*	transcriptional regulator ToxR	0.54	0.000178676
